# Hyponatremia upon PICU admission: a 10-year retrospective analysis of outcomes and independent prognostic value

**DOI:** 10.1186/s12887-026-06888-2

**Published:** 2026-04-21

**Authors:** Florian Weber, Christoph Quatember, H.-Ch. von Andrian, Michael K. Baumgartner, Nicolas Allgaier, Felicitas Ilg, Lea S. Samija, Peter Baumgarten, Johanna Messner, Melanie L. Conrad, Fabian B. Fahlbusch

**Affiliations:** 1 Interdisciplinary Pediatric Emergency Department, Faculty of Medicine, University of Augsburg, Augsburg, 86156 Germany; 2 Neonatology and Pediatric Intensive Care, Faculty of Medicine, University of Augsburg, Augsburg, 86156 Germany; 3 Department of Neurosurgery, Faculty of Medicine. Section of Pediatric and Adolescent Neurosurgery, University of Augsburg, Augsburg, 86156 Germany; 4 Institute of Microbiology, Infectious Diseases and Immunology, Charité- Universitätsmedizin Berlin, Corporate Member of Freie Universität Berlin, Humboldt- Universität zu Berlin, Berlin, 12203 Germany; 5 Department of Pediatrics and Adolescent Medicine, Faculty of Medicine, University of Augsburg, Stenglinstr. 2, Augsburg, 86156 Germany

**Keywords:** Hyponatremia, PICU admission, Inflammation, Infection, Seizures, Pediatric critical care, HAH

## Abstract

**Background:**

Hyponatremia is a common electrolyte disturbance in critically ill children that has been associated with increased morbidity and mortality. This retrospective, single-center study investigates the prevalence and clinical relevance of hyponatremia at the time of pediatric intensive care unit (PICU) admission, with a particular focus on its association with inflammatory markers and seizures.

**Methods:**

We conducted a retrospective observational cohort study of pediatric patients beyond the neonatal period who were admitted with hyponatremia (serum sodium ≤ 135 mmol/L) to a German tertiary mixed pediatric and neonatal ICU between January 2015 and October 2024. Among 5,595 admissions screened, 2,906 non-neonatal patients were eligible; 113 presented with hyponatremia upon admission. After excluding cases with type 1 diabetes, chronic diuretic therapy, or hospital-acquired hyponatremia (HAH), 64 patients remained for analysis. Clinical characteristics, inflammatory markers (CRP, IL-6, PCT), and neurological events were evaluated.

**Results:**

Among the 64 included children (median serum sodium 129.5 mmol/L), infections were the leading cause of admission. Hyponatremia frequently preceded the rise of inflammatory markers and showed no significant association with CRP, IL-6, or PCT. However, IL-6 and PCT were not routinely measured, limiting interpretability. Length of stay primarily reflected underlying disease severity and showed no correlation with sodium levels. Seizures occurred in 16.4% of patients, although a direct causal link to hyponatremia could not be confirmed. COVID-19–associated hyponatremia was identified in 11 cases, including presentations with pediatric inflammatory multisystem syndrome (PIMS) and encephalopathy.

**Conclusion:**

Hyponatremia at PICU admission appears to represent an early indicator of disease burden that is not consistently reflected by conventional inflammatory biomarkers. Its frequent occurrence in infectious and neurological conditions underscores the importance of early recognition and structured interdisciplinary communication across emergency, pediatric, and critical care teams. Prospective studies are needed to validate these findings and delineate disease-specific mechanisms.

## Introduction

### Hyponatremia as a marker of clinical severity in pediatric critical illness

Hyponatremia, defined as a serum sodium (SNa) concentration below 135 mmol/L, is among the most common electrolyte disorders in clinical practice and occurs with particularly high frequency in critically ill children [1]. Reported prevalence among pediatric intensive care unit (PICU) admissions varies widely across studies, ranging from approximately 15% to over 60% [1]. Hyponatremia has been consistently linked to adverse outcomes, including increased morbidity and mortality, with hyponatremic encephalopathy representing a major concern, particularly when SNa falls below 125 mmol/L [2]. In a Finnish cohort, the risk of neurological symptoms (OR: 1.8; *p* = 0.02) and death during hospitalisation (OR: 33; *p* = 0.001) of acutely ill children was markedly elevated among those with SNa < 130 mmol/L [3]. Hyponatremia is associated with neurological complications such as seizures, cerebral edema, and altered mental status [2, 4], as well as longer hospital stays and a higher likelihood of intensive care admission [1, 5]. Early recognition and management can reduce morbidity and shorten hospitalization. Nevertheless, hyponatremia remains frequently underrecognized as an early warning marker [2, 4], in part because it is unclear whether it directly contributes to excess mortality or merely reflects the severity of an underlying life-threatening condition [6].

### Determinants of hyponatremia in pediatric critical illness

The causes of hyponatremia in critically ill children are multifactorial, encompassing renal and extrarenal sodium losses, shifts in total body water, and free water retention mediated by arginine vasopressin (AVP) [7, 8]. These fluctuations are amplified in children due to their relatively high total body water, which predisposes them to rapid volume changes [7]. Non-osmotic AVP release - triggered by hypovolemia, pain, stress, or hypoxia - has long been recognized as a dominant mechanism of hyponatremia in pediatric illness. Inflammatory cytokines can also stimulate AVP secretion [9, 10], promoting dilutional hyponatremia even in the absence of osmotic or hemodynamic triggers [4, 11].

Hyponatremia has been documented across a range of pediatric inflammatory and infectious conditions [12], including bronchiolitis [5, 13–15], Kawasaki disease [10, 16, 17], multisystem inflammatory syndrome in children (MIS-C) or pediatric inflammatory multisystem syndrome (PIMS), COVID-19 infection [18–22], meningoencephalitis [23, 24] and human immunodeficiency virus (HIV-1) infection [25]. These associations suggest a broader interplay between inflammation, infection, and fluid regulation. Surgical pathologies, such as perforated appendicitis [26], can contribute through third spacing, systemic inflammation, and perioperative fluid shifts. Non-infectious neurologic and traumatic conditions, including head trauma [27], also predispose to hyponatremia, partly via stress-induced AVP release and altered intracranial dynamics.

Collectively, these mechanisms indicate that hyponatremia in pediatric critical illness often represents a marker of inflammatory and neurohormonal stress rather than a primary electrolyte imbalance.

### Hospital-Acquired Hyponatremia (HAH)

Hospital-acquired hyponatremia (HAH) remains a significant concern in pediatric care. Historically, the administration of hypotonic maintenance intravenous fluids amplified the effects of AVP-mediated water retention, contributing to neurological complications such as cerebral edema and seizures [2, 28–32]. These risks prompted the American Academy of Pediatrics (AAP) in 2018 to recommend isotonic maintenance fluids for hospitalized children aged 28 days to 18 years [33]. Children are particularly susceptible to symptomatic hyponatremia and cerebral edema due to their larger brain-to-skull ratio, especially under six years of age, when brain size nears adult proportions before skull growth is complete [4]. Even modest SNa declines can therefore be clinically significant. While HAH is an important source of morbidity, this analysis focuses exclusively on hyponatremia present at PICU admission, prior to the influence of hospital-based interventions.

### Study rationale and objectives

Hyponatremia at PICU admission, prior to exposure to fluid therapy, diuretics, or prolonged intensive care, may serve as a practical early marker of disease severity. Severe hyponatremia at presentation has been associated with longer PICU stays and a trend toward increased mortality [1, 34]. This study, conducted at a German tertiary care PICU between January 2015 and October 2024, examines whether admission sodium levels correlate with outcomes such as PICU length of stay and seizure incidence, and whether these associations are independent of inflammatory biomarkers including CRP, IL-6, and PCT. By focusing on hyponatremia present at admission, this study aims to clarify whether low sodium levels primarily reflect an inflammation-related neurohormonal response or function as an independent early indicator of physiological stress [11, 35, 36], while minimizing confounding by hospital-acquired factors such as fluid management or extended intensive care treatment.

## Methods

### Cohort

**Fig. 1:**
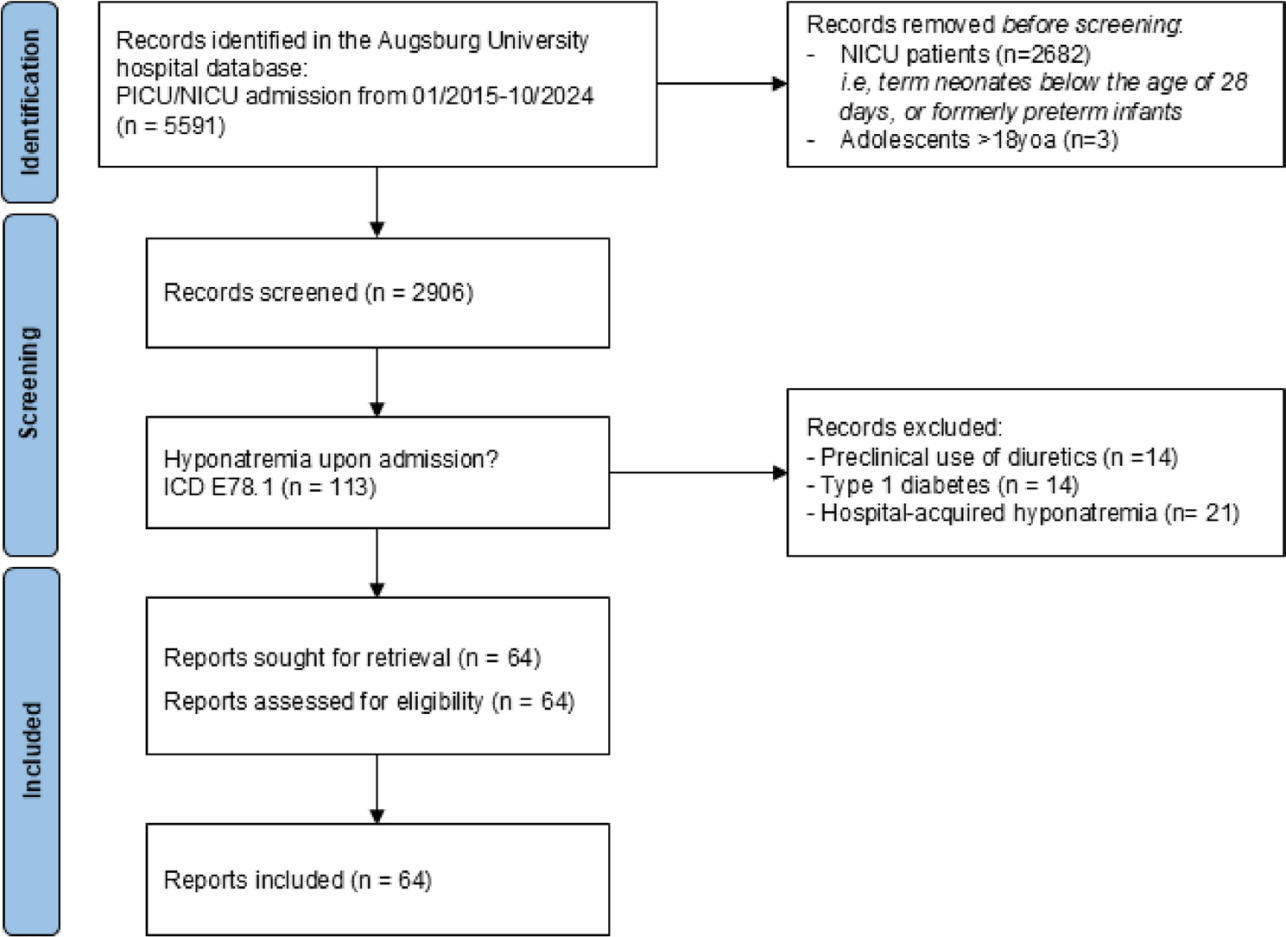
Study flow diagram showing inclusion and exclusion of patients. Legend: ICD = International Classification of Diseases 10th Revision, E87.1 = Hyponatremia

The study was based on a retrospective analysis of patient records from 01/2015 to 10/2024 at the Department of Pediatrics and Adolescent Medicine, Faculty of Medicine, University Hospital Augsburg, Germany, that houses a tertiary intensive care unit for neonatal and pediatric care as well as a tertiary interdisciplinary Pediatric Emergency Department (iPED). Inclusion criteria were: blood sodium levels ≤ 135mmol/L at the day of PICU admission, as indicated by International Classification of Diseases 10th Revision (ICD-10) code E87.1. Clinical data were obtained from the electronic patient management system (ORBIS, Deadalus, Germany). We focused on pediatric patients beyond the neonatal period (> 28 days of life) to avoid interference with translocation hyponatraemia, excluding preterm infants known for a prolonged period of high urinary losses of sodium and negative sodium balance [7, 37]. We further excluded patients with chronic diuretic therapy and those with type 1 diabetes mellitus, due to the frequent occurrence of complex electrolyte disturbances in this population, as well as cases of HAH [7, 38]. Figure 1 shows the flow diagram regarding inclusion and exclusion criteria of patients. 64 children were finally included in the study. We recorded the length of the PICU stay, duration of invasive ventilation if present, survival, infectious parameters and body temperature. CRP (mg/dl, lower limit of detection [LLOD] 0.21 mg/dl), IL-6 (pg/ml, upper limit of detection [ULOD] 50000 pg/ml), and PCT (ng/ml, ULOD 100 ng/ml) were measured in the hospital’s routine clinical laboratory. Inflammatory markers were evaluated exclusively at ICU admission to avoid potential confounding by hospital-acquired infections (HAI). For body temperature, the single measurement closest to ICU admission within a ± 12-hour window was recorded to ensure consistency with the assessment of inflammatory markers and to account for physiological fluctuations. As this was a retrospective analysis, the use of prehospital antipyretic medication could not be systematically assessed. Fever was defined as > 38.0 °C and hypothermia as < 36.0 °C.

### Data analysis

Descriptive statistics, including mean, standard deviation (SD), and median, were calculated to summarize the data. Due to the limited sample size (64 patients) and the heterogeneity in age and diagnoses, the study followed a primarily descriptive approach, restricting inferential analyses to exploratory correlations and group comparisons. For correlation analysis, only CRP and body temperature were included, as IL-6 and PCT data were incomplete for the majority of patients. Pearson correlation coefficients were calculated to explore potential linear associations between sodium, CRP, and body temperature, acknowledging that the study was not powered for hypothesis testing. A linear regression model was applied to assess whether CRP or temperature predicted sodium levels; this analysis was restricted to the subset of patients (*n* = 61) with complete data for all three parameters. For group comparisons between patients with severe hyponatremia (< 125 mmol/L) and those with sodium ≥ 125 mmol/L, Welch’s t-test was used to compare CRP and temperature, given unequal variances between groups. A p-value < 0.05 was considered statistically significant. Data are presented as mean ± SEM unless stated otherwise. All analyses and data visualization were conducted using GraphPad Prism version 10 (GraphPad Software Inc., La Jolla, USA), with a p-value of ≤ 0.05 considered indicative of a statistically notable trend rather than a confirmatory result.

## Results

### Cohort characteristics

Out of 2,906 patients screened, 113 were identified with hyponatremia upon admission. Among them, 12.4% had type 1 diabetes mellitus, and 12.4% were receiving diuretic therapy at the time of PICU admission, leading to their exclusion from the analysis. Hospital-acquired hyponatremia (HAH) was observed in 18.6% of the cases. After applying the exclusion criteria, a total of 64 children (26 females, 38 males) with hyponatremia at PICU admission were included in the study. The median SNa level was 129.5 mmol/L (range: 113–135 mmol/L). The median age was 6 years (range: 1–16 years), with 42.2% of patients being under the age of two. The majority of patients (71.9%, *n* = 45) were admitted directly to the PICU.

### Distribution of diagnoses and inflammatory parameters

**Fig. 2:**
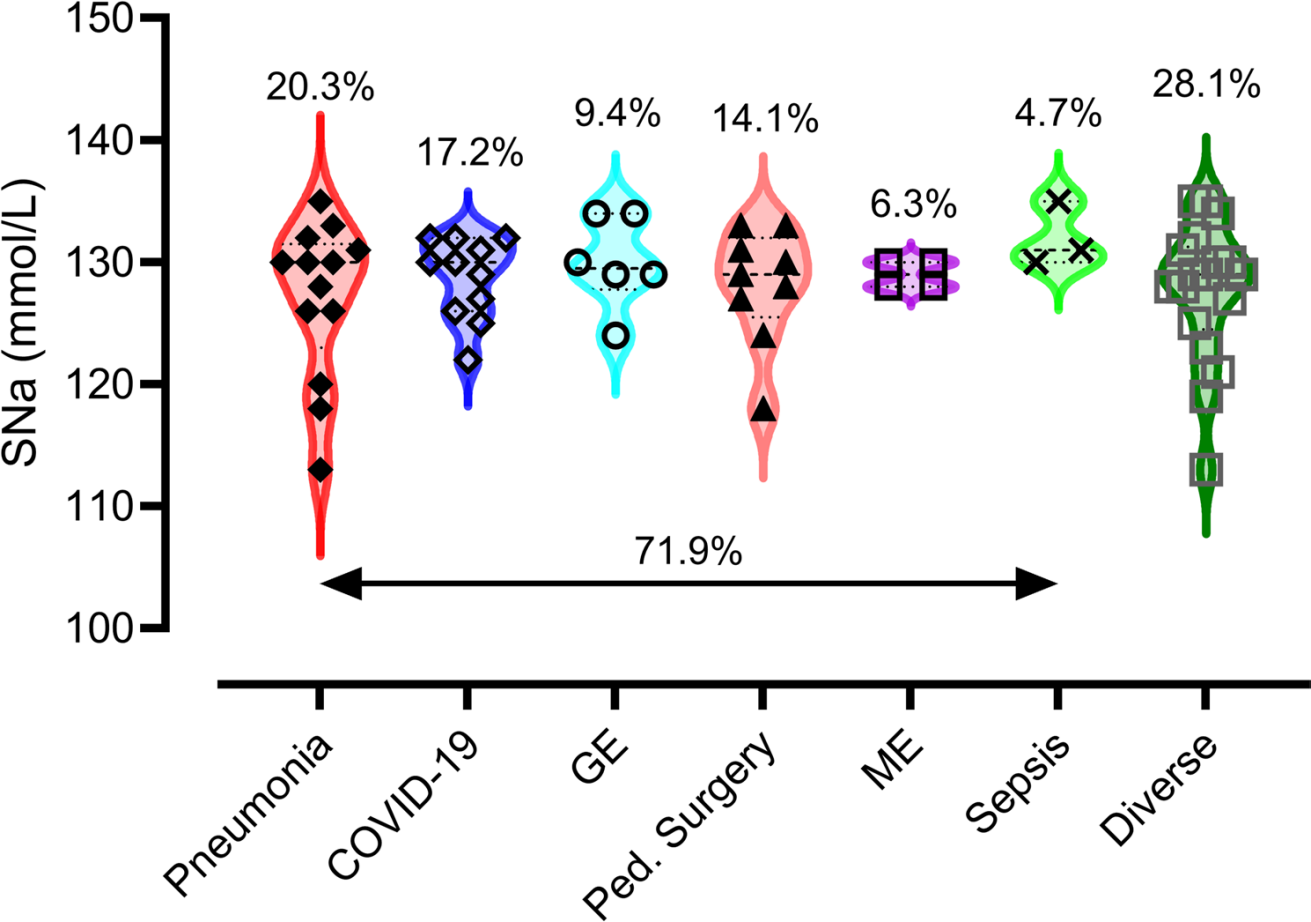
Disease conditions and hyponatremia at PICU admission. Legend: GE = Gastroenteritis, Ped. = Pediatric, ME = Meningoencephalitis. 71.9% were conditions associated with infections

Figure 2; Table 1 illustrate the distribution of admission diagnoses, duration of PICU stay, and associated inflammatory markers. C-reactive protein (CRP) was available for all patients, while interleukin-6 (IL-6) and procalcitonin (PCT) were only determined in 45.3% and 37.5% of patients, respectively (Table 1). A total of 71.9% of patients were diagnosed with infection-related conditions, including pneumonia (20.3%), COVID-19 (17.2%), gastroenteritis (9.4%), pediatric surgical emergencies (14.1%), meningoencephalitis (6.3%), and sepsis (4.7%). COVID-19-associated hyponatremia was observed between November 2020 and November 2023 (*n* = 11), including seven PIMS cases and two encephalopathy cases, one of which was fatal. Bronchiolitis did not appear as a separate category. Re-examination of all lower-airway infections in children ≤ 2 years identified four pneumonia cases (one *Moraxella catarrhalis*, three RSV), none of which met criteria for classic bronchiolitis. No cases of head trauma were identified. The diverse group comprised a heterogeneous set of non-infectious conditions (e.g., adrenal crisis, diabetes insipidus, VP-shunt dysfunction, hemolytic uremic syndrome, nephrotic syndrome, exocrine pancreatic insufficiency, feeding difficulties in infants with complex medical histories, recurrent sinus venous thrombosis, and syndromic terminal events) and a small number of miscellaneous infectious presentations (*n* = 4; e.g., pyelonephritis, pharyngitis, epidural abscess) that did not fit into the main diagnostic clusters. In the pediatric surgery subgroup, hyponatremia was most commonly associated with perforated appendicitis.

**Table 1:** Descriptive data analysis. Various primary diagnoses were associated with hyponatremia at PICU admission. The table further shows the duration of PICU stay (days) and the inflammatory markers. Hyponatremia: mild = 131–135 mmol/L; moderate = 125–130 mmol/L, and severe < 125 mmol/L. GE, gastroenteritis; ME, meningoencephalitis

Median (min-max)	Pneumonia	COVID-19	GE	Pediatric Surgery	ME	Sepsis	Diverse Cases
Sodium (mmol/L)	130 (113–135, *n* = 13)	130 (122–132, *n* = 11)	129.5 (124–134, *n* = 6)	129 (118–133, *n* = 9)	129 (128–130, *n* = 4)	131 (130–135, *n* = 3)	129 (112–135, *n* = 18)
Mild (*n* = 20)	4	4	2	3	0	2	5
Moderate (*n* = 33)	6	6	3	4	4	1	9
Severe (*n* = 11)	3	1	1	2	0	0	4
PICU days > 24 h	10.0 (2-162, *n* = 12)	3 (1–24, *n* = 11)	1 (1–5, *n* = 6)	4 (1–65, *n* = 8)	4 (1–12, *n* = 4)	8 (2–9, *n* = 3)	4 (1–33, *n* = 16)
CRP (mg/dl)	2.68 (0.21–46.2, *n* = 13)	10.2 (0.21–39.6, *n* = 11)	6.44 (0.21–25.3, *n* = 6)	18.79 (0.38–35.41, *n* = 9)	25.6 (6.0-33.53, *n* = 4)	10.02 (7.12-29.0, *n* = 3)	0.52 (0.21–23.2, *n* = 18)
IL-6 (pg/ml)	21.5 (9.8–8760, *n* = 9)	1260 (40.4-50000, *n* = 5)	9.0 (7.5–10.5, *n* = 2)	48.7 (9.59–151, *n* = 3)	6186 (4949–7423, *n* = 2)	2027 (1564–2490, *n* = 2)	14.5 (9.5–80.6, *n* = 6)
PCT (ng/ml)	5.45 (0.16–100, *n* = 6)	31.0 (9.4–100, *n* = 7)	3.7 (*n* = 1)	9.7 (0.54-12, *n* = 3)	38 (*n* = 1)	70 (20–100, *n* = 3)	0.39 (0.37–100, *n* = 3)

Following the implementation of isotonic maintenance fluid recommendations in 2018 [33], we did not observe a major shift in the etiologic profile of hyponatremia at PICU admission. Before 2018, 19 hyponatremic admissions were recorded over four years (4.75 cases per year), compared with 37 non–COVID-19 cases over five years (7.4 cases per year) thereafter. Apart from this moderate increase in annual case numbers, the distribution of infection-related and neurological diagnoses remained largely unchanged. The only notable difference was a higher proportion of pediatric surgical cases in the later period (5% before 2018 vs. 21.6% after 2018).

### Association with inflammatory markers and clinical outcomes

**Fig. 3:**
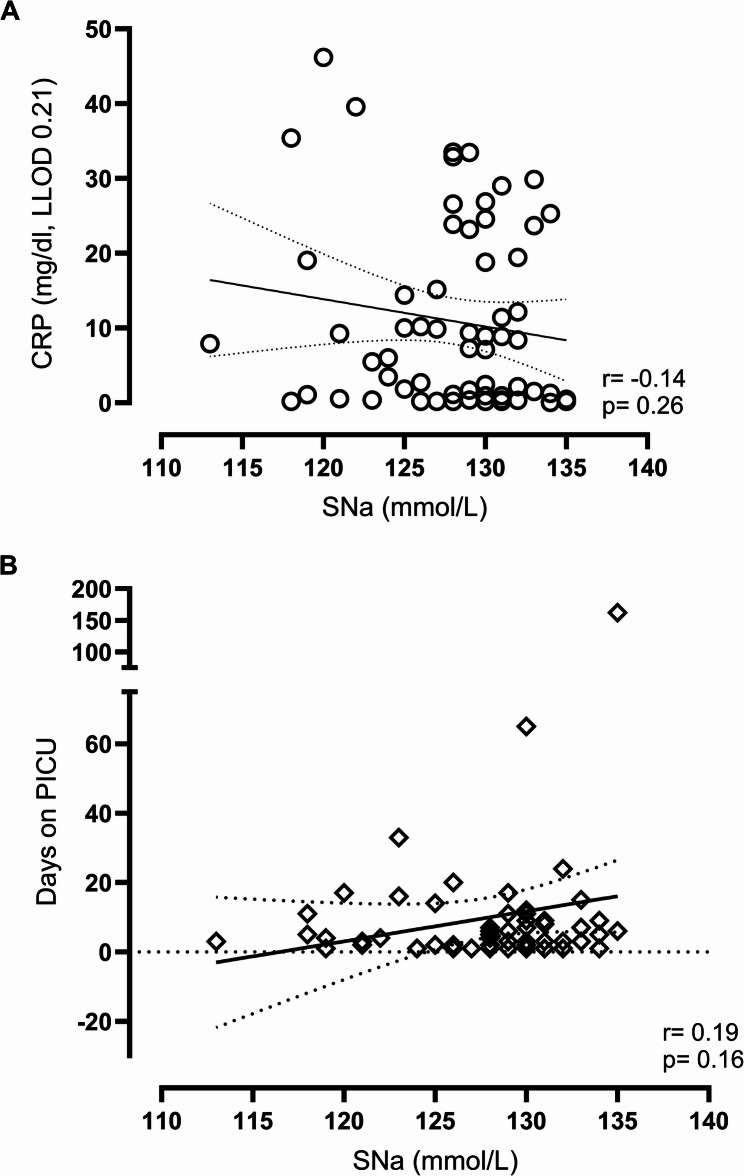
Pearson correlation analysis. **A** CRP during the admission period (y-axis) vs. SNa levels at PICU admission (x-axis). Legend: LLOD = Lower Limit of Detection. **B** Number of ICU days (y-axis) vs. SNa levels at PICU admission (x-axis)

There was no significant correlation between sodium levels and inflammatory markers including CRP (Fig. 3A; *r*= -0.14, *p* = 0.26), IL-6, or PCT (*r* = 0.13, *p* = 0.51 and *r*= -0.28, *p* = 0.18, respectively). Similarly, no association was found between hyponatremia and PICU length of stay (Fig. 3B; *r* = 0.19, *p* = 0.16) or duration of mechanical ventilation (*data not shown*).

Among diagnostic groups, pneumonia cases exhibited the longest hospital stays (28.3 ± 16.8 days; median ventilation duration 141 h), followed by COVID-19–associated cases (10.2 ± 4.3 days; median ventilation duration 121 h). Respiratory support was required in 19 children overall, including 5 with COVID-19 and 9 with pneumonia. At PICU admission, invasive ventilatory support was documented in 2/5 COVID-19 cases and 4/9 pneumonia cases. Serum sodium levels did not differ between children requiring invasive respiratory support at admission (*n* = 6; 126.6 ± 2.50 mmol/L) and those who required invasive ventilation later during their PICU stay (*n* = 3; 124.7 ± 2.60 mmol/L; *p* = 0.6). The pneumonia group included two children with pre-existing tracheostomies and one patient transferred to an external ECMO center. During the PICU course, invasive ventilation was required in 4/5 children with COVID-19 and in 3/9 with pneumonia, while non-invasive modalities were used at some point in nearly all cases. Given the small sample sizes, these descriptive patterns should be interpreted cautiously.

**Fig. 4:**
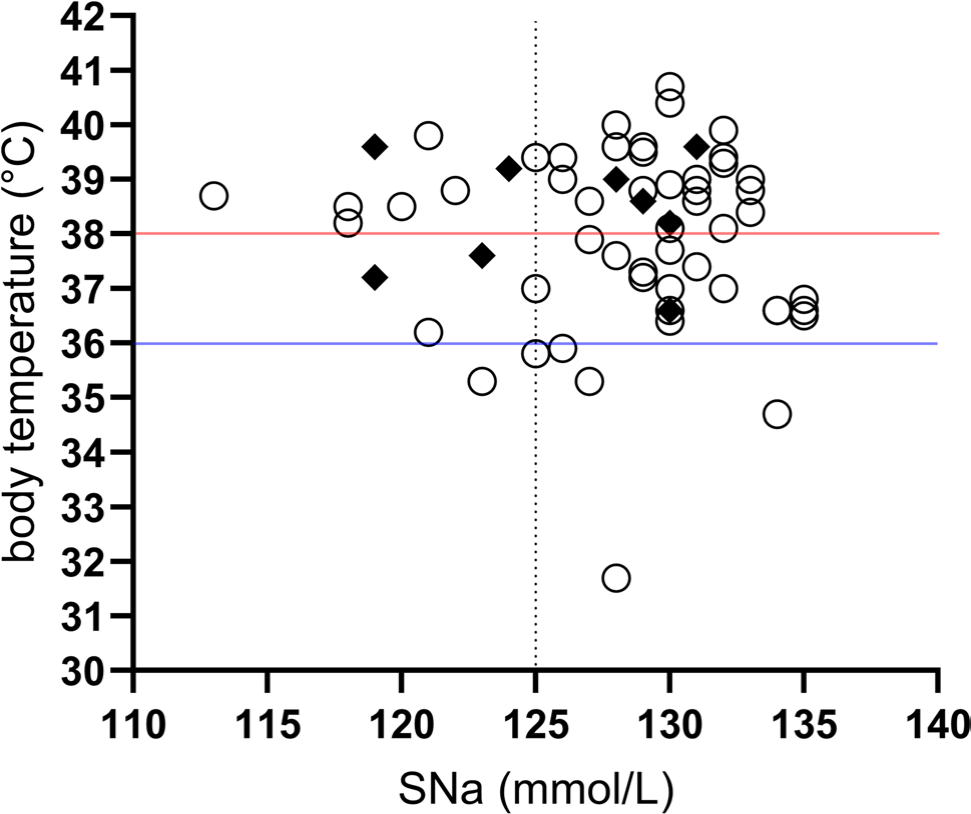
Body temperature versus serum sodium (SNa) at PICU admission. The x-axis represents SNa (mmol/L), and the y-axis shows body temperature (°C) measured within 12 h of admission. This plot visualizes the relationship between temperature, sodium levels, and seizure occurrence in the cohort (*n* = 61/64 paired datasets). Horizontal lines denote fever (> 38 °C, red) and hypothermia (< 36 °C, blue). A vertical dotted line marks the 125 mmol/L threshold, below which approximately 50% of pediatric patients are reported to be at elevated risk to develop hyponatremic encephalopathy [2]. Patients with seizures (*n* = 9/11, complete paired datasets) are indicated as diamonds; those without seizures are shown as circles

We additionally evaluated whether fever at admission - used as an indicator of systemic inflammation - was associated with hyponatremia. Among 61 patients with available data, body temperature ranged from 31.7 °C to 40.7 °C (median: 38.3 °C). Most patients were febrile (*n* = 35; 38.1–40.7 °C, median: 39.0 °C), while six exhibited significant hypothermia (31.7–35.9 °C, median: 35.3 °C); only 32.8% were normothermic. Body temperature showed a trend toward positive correlation with CRP (*r* = 0.25, *p* = 0.054), which could be consistent with systemic inflammatory activity. However, no significant associations were observed between SNa and CRP (*r* = -0.14, *p* > 0.1) or between sodium and body temperature (*r* = -0.08, *p* > 0.1). Linear regression analysis likewise indicated that neither CRP nor temperature predicted sodium levels (R² = 0.02 and 0.06, respectively). Comparison of patients with severe hyponatremia (< 125 mmol/L, *n* = 11) and those with sodium ≥ 125 mmol/L (*n* = 53) revealed no significant differences in CRP (16.25 ± 5.0 vs. 13.19 ± 1.61 mg/dL, *p* = 0.47) or body temperature (37.9 ± 0.23 vs. 38.1 ± 0.34 °C, *p* = 0.68). Figure 4 shows the patient distribution with regard to SNa, body temperature and seizure events.

### Seizure events

**Fig. 5:**
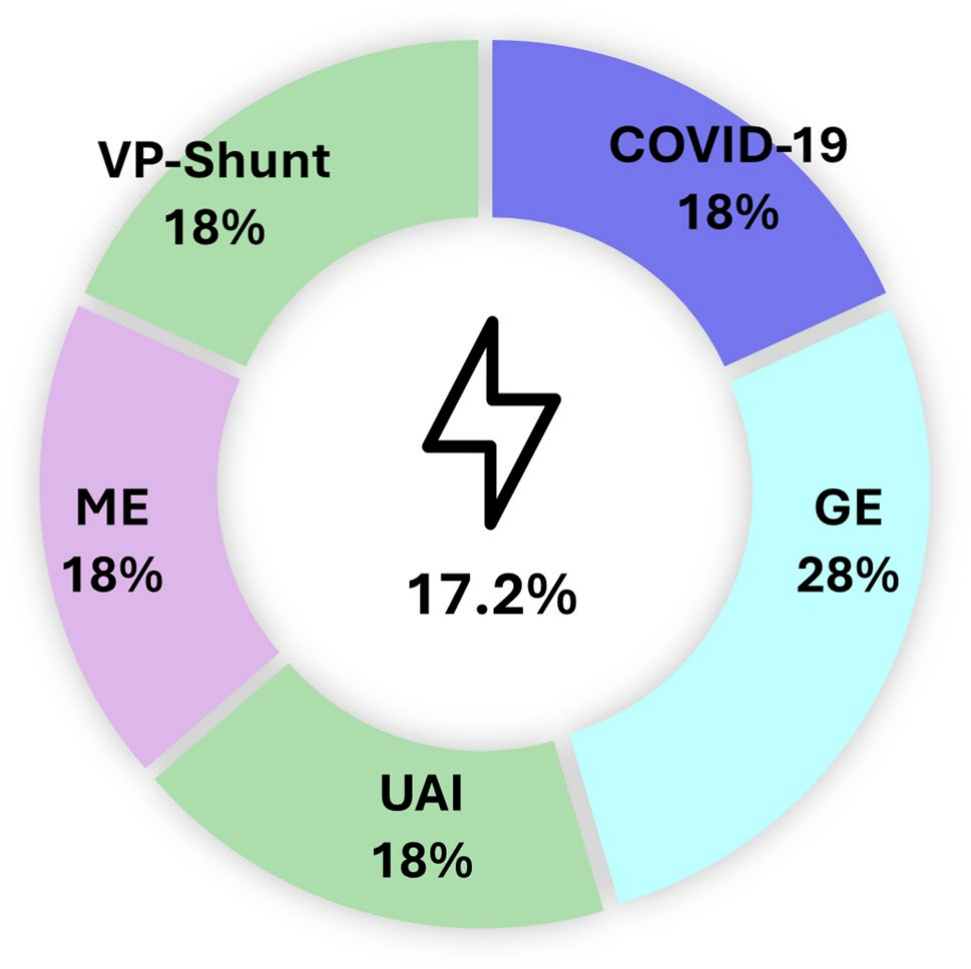
Patients with seizures in the context of accompanying hyponatremia (AHN) (*n* = 11/64). One patient on anticonvulsive therapy for brain tumor and status epilepticus due to VP-shunt insufficiency (total *n* = 2). Two cases of COVID-19-associated encephalopathy (one fatal) and pneumococcal encephalitis, each. Three cases of GE. Febrile seizures (*n* = 3): 1 GE, 2 UAI. Legend: GE, gastroenteritis; UAI, febrile upper airway infection; ME, meningoencephalitis; flash icon symbolizing seizure (from: www.flaticon.com/authors/ambar)

Seizures requiring therapeutic intervention were documented in 17.2% of patients (11/64), with a mean age of 4 years (range 1–14); notably, 8 of these 11 children were younger than two years. Two of the older children had underlying organic brain disorder (astrozytoma - age 9 and spina bifida - age 14) and another suffered from one COVID-19-associated encephalopathy. All documented seizure events occurred prior to hospital admission, not on the PICU itself. The mean SNa concentration upon PICU admission among patients with seizures was 125.7 mmol/L. Although seizure events were clinically frequent in our cohort, no statistically significant association with hyponatremia could be demonstrated. The cases were distributed across various diagnostic categories (Fig. 5). In total, three febrile seizures were observed - one associated with gastroenteritis and two with upper airway infections. Only one patient had received anticonvulsant therapy prior to admission (astrocytoma).

### Mortality

The overall PICU mortality rate in the cohort was 4.7% (3/64). The deaths resulted from pneumonia in a child with mucopolysaccharidosis type II (palliative care), and cerebral edema with seizures in a COVID-19 case with pneumonia. One infant was admitted after an external suspected life-threatening event (sudden infant death syndrome, SIDS) and deteriorated rapidly after PICU arrival, with the fatal outcome reflecting the severity of the underlying condition. In none of the cases was hyponatremia identified as a direct contributing factor.

## Discussion

### Overview of main findings

This retrospective, single-center study investigated hyponatremia at PICU admission over a ten-year period in a tertiary pediatric acute care center. Sixty-four children with serum sodium < 135 mmol/L (median 129.5 mmol/L) were included, most of whom (71.9%) were admitted with infection-related conditions such as pneumonia, COVID-19, gastroenteritis, meningitis, or sepsis. Hyponatremia did not correlate with inflammatory markers (CRP, IL-6, PCT), PICU length of stay, or duration of mechanical ventilation. Seizures occurred in 16.4% of patients (mean SNa 125.7 mmol/L) but were documented only prior to PICU admission. Overall mortality was 4.7%, with no death directly attributable to hyponatremia. Taken together, these results provide a coherent picture of hyponatremia as an early feature of disease burden rather than a marker of downstream inflammation. This supports the concept that hyponatremia may arise early in the course of critical illness and can precede conventional inflammatory responses.

### Hyponatremia and infection-related mechanisms

Over 70% of hyponatremic cases of our pediatric cohort were linked to infectious diseases upon PICU admission, most commonly pneumonia, COVID-19, and sepsis. This mirrors previous reports identifying infection as a major driver of dysnatremia in pediatric critical illness. However, our findings diverge from studies such as Tagarro et al. [39] and others [13], which observed a negative correlation between sodium levels and inflammatory markers (CRP, PCT) in community-acquired pneumonia (CAP). We did not observe a correlation between SNa levels and CRP, IL-6, or PCT, suggesting that admission hyponatremia may reflect an early physiological disturbance not captured by these markers. Similar patterns have been reported in bronchiolitis [5, 13–15], Kawasaki disease [10, 16, 17], MIS-C/ PIMS and COVID-19 [18–22], and meningoencephalitis [23, 24] where hyponatremia often co-occurs with systemic inflammation but not consistently with conventional biomarkers. This suggests that alternative mechanisms, such as IL-1- and TNF-mediated renal salt wasting, as proposed by Watanabe [40, 41] and Eisenhut [42], may play a more prominent role in inflammation-associated hyponatremia than IL-6 or CRP.

Perforated appendicitis, a frequent diagnosis within our surgical subgroup, was particularly associated with low SNa levels. In these cases, hyponatremia likely reflects the severity of the condition, driven by third spacing, systemic inflammation, and perioperative fluid shifts [26]. Once perforation occurs, the ensuing infectious burden and progression toward systemic inflammatory response syndrome (SIRS) or sepsis further amplify these effects [30]. Consequently, low SNa at admission may serve as an early clinical trait signalling severe disease, and warranting heightened monitoring and prompt coordination between the iPED, pediatric surgery, and the PICU to ensure timely intervention and optimized care.

Neurological complications, including VP shunt dysfunction and encephalitis, commonly observed in our cohort, are also recognized contexts for hyponatremia due to stress-induced AVP release and, in some cases, cerebral salt wasting [4, 43, 44].

These diverse mechanisms highlight that hyponatremia at PICU admission represents a heterogeneous phenomenon - frequently infection-related, but at times driven by neurohormonal influences or altered renal sodium handling. Our findings indicate that, at the time of hospital entry - whether first seen in the iPED or directly admitted to the PICU - hyponatremia might not be consistently reflected by standard inflammatory markers and must therefore be promptly recognized and contextualized to guide early triage and management.

### Divergence from prior literature

Several studies have demonstrated associations between hyponatremia, inflammatory markers, prolonged PICU stays, and adverse outcomes [9, 11, 45]. Recent studies have continued to highlight the clinical relevance of hyponatremia in pediatric acute care, showing that it remains the most common electrolyte disturbance in critically ill children - particularly in neurologic and neurosurgical conditions [46] - that even mild hyponatremia is associated with increased morbidity and higher admission rates in emergency settings [34], and that hypotonic maintenance fluids continue to contribute to hyponatremia and prolonged hospitalization in intensive care [47].

Our findings did not replicate these associations. The differences may be attributable to multiple factors: Our cohort primarily consisted of acute admissions rather than patients with prolonged ICU exposure, reducing the potential confounding impact of HAH. Sodium levels were measured uniformly at admission, whereas other studies may have captured later derangements influenced by therapeutic interventions or cumulative inflammatory responses. Although the cohort was relatively small and heterogeneous, this reflected the stringent recruitment criteria applied to minimize confounding factors and ensure a well-defined study population. Nevertheless, the incomplete measurement of IL‑6 and PCT (with CRP as the only marker consistently available) may have reduced the ability to detect more subtle or cytokine‑specific associations. This gap might reflect adaptive diagnostic practices, in which CRP is widely available and inexpensive, while IL-6 and PCT are typically reserved for more severe or complex cases.

Following the implementation of isotonic maintenance fluid recommendations in 2018 [33], we did not observe a major shift in the etiologic profile of hyponatremia at PICU admission. The observed temporal patterns (i.e., COVID-19 pandemic and increase in pediatric surgical cases after 2018) are unlikely to reflect effects of the 2018 fluid guideline [33] and more likely represent natural variation in case mix over time.

Gultekingel et al. identified fever as a relevant confounder for hyponatremia in acute bronchiolitis [14]. As our cohort did not include classic bronchiolitis cases, comparability is limited; however, the concept that fever may modulate sodium levels in single-etiology cohorts remains informative. In our heterogeneous PICU population, body temperature showed only a weak trend toward association with CRP and no association with serum sodium. This suggests that, in mixed critical illness, febrile responses and hyponatremia are not closely coupled and likely arise through partly independent mechanisms. The incomplete availability of IL-6 and PCT restricted further exploration of cytokine-specific pathways.

### Neurological risk and implications for emergency care

Seizures were documented in 16.4% of hyponatremic patients, with a mean SNa level of 125.7 mmol/L. Notably, all seizure events occurred before hospital admission, underscoring the clinical relevance of hyponatremia for emergency care providers. Eight of the eleven seizure cases involved children under two years of age, a group particularly vulnerable to cerebral complications due to their disproportionately large brain-to-skull ratio [4], which leaves little reserve to accommodate swelling. Although a causal relationship between hyponatremia and seizures could not be confirmed, the high seizure prevalence in our cohort underscores the need for vigilant sodium monitoring and prompt recognition of neurological risk across pre-hospital, emergency, and inpatient care. Clear communication of this risk between frontline teams, pediatric specialists of the iPED, and the PICU is essential to ensure timely intervention, particularly for children under six years of age and those with SNa levels below 125 mmol/L, a threshold below which approximately 50% of pediatric patients are reported to be at elevated risk to develop hyponatremic encephalopathy [2].

### Limitations

The study’s retrospective, single-center design limits causal inference and generalizability. IL-6 and PCT were available in only 45.3% and 37.5% of cases, respectively, permitting robust correlation and regression analyses only with CRP. A normonatremic comparison group was not included, as the study was designed to descriptively assess children presenting with hyponatremia at PICU admission in a heterogeneous clinical setting, where retrospective matching would have introduced substantial confounding. The diversity of underlying diagnoses may have obscured condition-specific associations, and subgroup sensitivity analyses were not feasible because individual diagnostic groups contained too few patients for meaningful comparison. Because a proportion of patients were transferred from other hospital departments or from externally, pre-PICU clinical management (including fluids, analgesia/sedation, and antipyretic therapy) could not be fully reconstructed. This introduces potential variability in baseline physiology at PICU entry. Future prospective studies should therefore consider restricting inclusion to direct PICU admissions to minimize these pre-admission influences. Standardized severity scores such as PELOD-2 [48] were not established in our PICU during the study period, and retrospective reconstruction was not feasible within the constraints of the available data. In addition, ADH levels were not available because they are not routinely measured in our clinical setting. Measurements of ADH or fractional excretion of sodium (FeNa), as proposed by others [39, 41], may have further clarified renal mechanisms contributing to hyponatremia.

### Outlook and clinical implications

Hyponatremia present at PICU admission, before the influence of fluid therapy or prolonged intensive care, may serve as an early marker of disease burden - potentially independent of systemic inflammation as captured by conventional markers, or alternatively reflecting pathways not measured in this study. While not directly predictive of outcomes such as mortality or ventilation duration in this cohort, its frequent association with infectious etiologies and pre-admission seizures underscores the importance of structured interpretation and early communication across emergency, pediatric, and critical care teams. Sodium levels should be contextualized within the broader clinical picture and should inform triage and monitoring decisions from the earliest stages of care.

Future studies should explore whether alternative mediators such as IL-1, TNF, and renal indices (e.g., FeNa) better explain inflammatory hyponatremia than CRP or IL-6 and whether targeted management strategies can reduce its clinical impact. As highlighted by Sterns [6], controlled trials are essential to determine whether hyponatremia itself contributes to adverse outcomes or primarily reflects underlying disease severity, and to develop evidence-based approaches for reducing morbidity and hospitalization in this vulnerable population.

## Data Availability

All data supporting the findings of this study are included within the manuscript. Further data or details are available from the corresponding author upon reasonable request.

## Abbreviations

AVP: Arginine Vasopressin
AHN: Accompanying Hyponatremia
CNS: Central Nervous System
CRP: C-reactive protein
HAH: Hospital-acquired hyponatremia
ICD-10: International Statistical Classification of Diseases and Related Health Problems, 10th Revision
ICU: Intensive Care Unit
IL: Interleukin
iPED: Interdisciplinary Pediatric Emergency Department
LLOD: Lower limit of detection
MIS-C: Multisystem inflammatory syndrome in children
NICU: Neonatal Intensive Care Unit
PCT: Procalcitonin
PICU: Pediatric Intensive Care Unit
PIMS: Pediatric Inflammatory Multisystem Syndrome
SIADH: Syndrome of Inappropriate Antidiuretic Hormone Secretion
SNa: Serum sodium
ULOD: Upper limit of detection
